# Effect of in vitro gastrointestinal digestion on the antibacterial activity of bioactive dairy formulas supplemented with lactoferrin against *Cronobacter sakazakii*

**DOI:** 10.1007/s10534-022-00459-5

**Published:** 2022-11-06

**Authors:** Inés Abad, Laura Serrano, Dimitra Graikini, María Dolores Pérez, Laura Grasa, Lourdes Sánchez

**Affiliations:** 1 Departamento de Producción Animal y Ciencia de los Alimentos. Facultad de Veterinaria, Universidad de Zaragoza, Saragossa, Spain; 2 Instituto Agroalimentario de Aragón IA2 (UNIZAR-CITA), Saragossa, Spain; 3 Departamento de Farmacología, Fisiología y Medicina Legal y Forense. Facultad de Veterinaria, Universidad de Zaragoza, Saragossa, Spain

**Keywords:** Lactoferrin, *Cronobacter sakazakii*, Bioactive peptides, Whey, Buttermilk, Technological treatments

## Abstract

Milk is a source of proteins with high nutritional value and relevant biological activities. Bioactive milk proteins, like lactoferrin, are important for newborn development and can also be used as ingredients in functional products to improve health. Lactoferrin is essential in infant’s diet, since protects against infections and promotes immune system maturation. Bovine lactoferrin is used to supplement formula milk in order to strengthen baby’s defences against some pathogenic bacteria. Thus, lactoferrin supplemented formula can be a barrier against emergent pathogens, such as *Cronobacter sakazakii*, which has caused great concern in the last few years. Milk proteins generate bioactive peptides in the digestion process, and it is known that industrial processing can modify their susceptibility to digestion. Treatments such as heating have been shown to denature whey proteins and make them more easily digestible. Therefore, the aim of this study was to analyze the effect of technological treatments and gastrointestinal digestion on the antibacterial activity against *C. sakazakii* of proteins present in dairy formulas supplemented with lactoferrin. Commercial bovine lactoferrin has been shown to have antibacterial activity against *C. sakazakii*, both in the native state and after static in vitro gastrointestinal digestion. In addition, the digests obtained from dairy formulas subjected to technological treatments, either homogenization or pasteurization, have higher antibacterial activity than non-treated formulas. The release of low molecular weight peptides during the in vitro gastric digestion is probably the cause that would explain the enhanced antibacterial activity of the digested dairy formulas.

## Introduction

According to the Codex Alimentarius (1999), milk is defined as the normal mammary secretion obtained from dairy animals, without any addition or extraction, intended for consumption as liquid milk or for processing. Similarly, according to Regulation (EC) No. 853/2004, milk is the secretion of the mammary glands of animals that has not been heated to a temperature above 40 °C or subjected to a treatment with equivalent effect. In any case, milk intended for commercialisation must be subjected to pasteurization or sterilization to eliminate all pathogenic microorganisms. Furthermore, milk can be subjected to other technological treatments, such as homogenization, which is usually applied to reduce milk fat globule size. In addition to ensuring dairy product safety, heat treatments also extend their shelf life (Claeys et al. 2013).

Milk is characterized by being a very complete food and it is considered a basic element in the diet. For this reason, there is a great demand for milk and dairy products. The main components of milk are protein, fat, lactose and minerals. The composition of milk varies among species and within the same species can also vary considerably depending on breed, stage of lactation, milking interval, type of feeding and climate (Roy et al. 2020). The major protein of most mammal milks is casein. This protein is only found in milk and it has a great biological value thanks to its content in essential amino acids (Fox et al. 2015). In addition to caseins, milk contains other proteins in the fraction called whey, which is obtained after acid or enzymatic precipitation of caseins, or by separation from casein by membrane filtration (Pires et al. 2021). The major whey proteins are β-lactoglobulin (β-LG), α-lactalbumin (α-LA), immunoglobulins, bovine serum albumin, lactoferrin (LF) and lactoperoxidase (Madureira et al. 2007).

Fat is present in milk as emulsified particles called fat globules, which are covered by a membrane known as the milk fat globule membrane (MFGM) and is mainly composed of proteins, phospholipids, cholesterol and glycerides (Manoni et al. 2020). The main proteins of MFGM are mucin, lactadherin, butyrophilin, xanthine oxidoreductase (XO) and adipophilin (Riccio 2004). Several health promoting effects have been proposed for MFGM, such as anticarcinogenic, antimicrobial, anti-inflammatory and anticholesterolemic activities (Manoni et al. 2020). However, the MFGM can be affected by technological treatments applied to milk, such as heat treatment, refrigeration or freeze-drying, losing or minimizing its beneficial effects (Singh 2006).

Most dietary proteins, and in particular milk proteins, produce bioactive peptides by the action of digestive enzymes during gastrointestinal digestion and milk fermentation. Once released, they are directed to the intestinal lumen, passing across the epithelium and reaching the systemic circulation to interact with target receptors (Tidona et al. 2009). These bioactive peptides can trigger physiological effects that influence body functions and consequently, impact positively on health. They exert functions such as antithrombotic, antihypertensive, antimicrobial and antiviral among others (Madureira et al. 2010).

In recent decades, a great interest in antimicrobial peptides has emerged, as an alternative to antibiotics. They consist of less than 100 amino acids and their composition includes positively charged residues, such as lysine, arginine and histidine, and a high amount of hydrophobic residues (Browne et al. 2020). Peptides with antimicrobial characteristics interact specifically with bacterial membranes, creating transmembrane pores and altering the osmotic gradient and consequently, membrane permeability (Hartmann et al. 2010). A great number of milk-derived bioactive peptides with interesting activities have been identified. Specifically, LF contains some antimicrobial peptides, such as lactoferricin and lactoferrampin. In addition, several bioactivities have been attributed to peptides from other milk proteins, such as caseins, β-LG or α-LA (Théolier et al. 2014).

Breast milk is the best source of nutrition for the newborn, as it contains a large number of bioactive agents. However, breast-feeding is not always possible, so it is important to have high-quality infant formula available. Milk mainly used as a base to make infant formulas is cow’s milk, although the demand for goat’s milk is increasing recently for this purpose. In addition, other ingredients are added to resemble mother’s milk as much as possible (Maathuis et al. 2017). However, the composition of breast milk differs quantitatively and qualitatively from cow’s milk. For this reason, the average nutrient content of breast milk is taken as a reference to elaborate infant formula (He et al. 2022). Furthermore, the fat of cow’s milk is replaced by plant oil blends to compensate for the deficit of unsaturated fatty acids and make it more similar to human milk fat (Pan et al. 2022). In addition, fat globules in cow’s milk are quite uniform in size, with a maximum of 4 µm (Bourlieu et al. 2015). However, human milk contains smaller fat globules, especially in the early stages of lactation, with a maximum size of 1.8 µm in colostrum (Rüegg and Blanc 1981). For this reason, homogenization and pasteurization are used to treat and stabilize infant formulas, reducing the size of cow’s milk fat globules (0.2–0.6 µm) and making them more similar to those of breast milk (Bourlieu et al. 2015). In these processes, a membrane composed of caseins and whey proteins is formed, thus maintaining milk fat globule structure (Pan et al. 2022). These technological treatments also modify the effect of digestion on dairy preparations, as it has been reported a higher release of fatty acids and a higher proteolysis of caseins in processed milk by homogenization or pasteurization (Bourlieu et al. 2015).

There are different processes to elaborate infant formula; some are based on mixing powder components in hygienic conditions and others on reconstituting powder ingredients, applying heat treatment and spray drying (Watson 2022). In any case, powder formulas are not a sterile product, and inadequate conditions of production, storage and handling can be a risk to infant health (Vargas-Leguás et al. 2009). For this reason, powdered milk products must be stored in a dry place, at temperatures below 20 °C and with a maximum relative humidity of 70%. In addition, it is recommended for powder milk to be consumed within a maximum period of one month once the container is opened, and to be administered immediately once reconstituted or keep in refrigeration no more than 24 h (Vargas-Leguás et al. 2009).

An emergent opportunistic pathogen that has caused great concern in the last few years has been *C. sakazakii*, as it has been associated to consumption of infant powder formula. To avoid and reduce the risk of infection by this pathogen, special care has been recommended in the handling and storage of powder formulas (Drudy et al. 2006). Although *C. sakazakii* causes illness in all age groups, its effects are most severe in children, and specially in low-weight babies, causing diseases like meningitis, necrotizing enterocolitis and sepsis in preterm and full-term infants (Henry and Fouladkhah 2019).

In any case, milk and dairy products are a source of nutrients and defensive factors not only for the newborn, but also for children and adults (Park and Nam 2015). Therefore, the main objective of this study was to evaluate the antibacterial activity against *C. sakazakii* of LF alone and in dairy formulas containing other milk bioactive proteins. The effect of technological treatments, such as homogenization or pasteurization, and of gastrointestinal digestion on the antibacterial activity of dairy formulas supplemented with LF have also been studied.

## Material and methods

### Obtaining dairy fractions

Raw bovine milk was provided by the dairy company Villacorona (El Burgo de Ebro, Spain), and processed at the Food Science and Technology Pilot Plant of the University of Zaragoza, located at the Veterinary Faculty. Previously to processing, milk pH, acidity, fat percentage, and alkaline phosphatase and lactoperoxidase activities were assessed. Milk was heated at 45–50 °C for 30 min in a cheese vat and skimmed in a skimming centrifuge model ARR-DES 125 (Suministros Químicos Arroyo, Santander, Spain). Skim milk was subjected to a second centrifugation to obtain the maximum amount of cream.

Then, skim milk was heated at 35 °C in a 25 L cheese vat and 30% CaCl_2_ was added into milk at dilution 1:8000 (v/v). Next, bovine rennet was added to milk at dilution 1:15,000 (v/v), evenly distributed and incubated for 1 h. After achieving casein coagulation, the curd was cut with lyres and whey was drained, filtered, lyophilized and kept at − 20 °C for later use.

Cream containing 43% of fat, previously obtained from raw milk as described above, was used to obtain buttermilk. Cream was maintained at 4 °C overnight, and then it was subjected to mechanical stirring with a Phillips Cucina mixer (Philips, Amsterdam, The Netherlands). This process was carried out until phase inversion took place, thus obtaining butter grains, which were formed by the agglomeration of milk fat globules, allowing the release of buttermilk. Buttermilk was filtered through cheese cloth and glass wool to remove the remaining butter grains, lyophilized and kept at − 20 °C.

### Preparation of samples and dairy formulas

Several dairy formulas were prepared to be subjected to static in vitro gastrointestinal (GIT) digestion and to subsequent evaluation of their antimicrobial activity against *C. sakazakii*. Commercial bovine LF used to supplement dairy formulas was donated by the company Tatua Nutritionals (Morrinsville, New Zealand).

Six dairy formulas were elaborated, based on whey or buttermilk and supplemented with LF and MFGM. Formula 1 (F1) was composed of whey (0.068 g/mL), LF (10 mg/mL) and MFGM obtained from the centrifugation at 40,000 g for 30 min at 4 °C of a volume (in 1:1 ratio with whey) of non-homogenized buttermilk. Formula 2 (F2) was made based on non-homogenized buttermilk, supplemented with LF (10 mg/mL) and MFGM, obtained as in F1, from a volume of non-homogenized buttermilk (in 1:1 ratio with buttermilk). Formulas 3 and 4 (F3 and F4) were prepared with the same composition as F1 and F2, respectively, but buttermilk was previously subjected to one-phase homogenization at 250 bar. Formulas 5 and 6 (F5 and F6) were also prepared with the same composition as F1 and F2, respectively, but were subjected to a pasteurization process at 72 °C for 20 s.

For thermal treatment, F5 and F6 were aliquoted into 1 mL vials and a thermal probe connected to a data logger (Almeno 2409, Ahlborn, Ilmenau, Germany) was placed inside one vial for temperature control. First, two water baths (Unitronic 200 and Precisterm S-138, both from J.P. Selecta, Barcelona, Spain) were tempered at 60 °C and 72 °C, respectively. The samples were introduced into the first bath at 60 °C and, upon reaching that temperature, they were transferred to the bath at 72 °C, where they were maintained for 20 s once they reached that temperature. After pasteurization, samples were cooled down immersing them into ice and afterwards, stored at − 20 °C until use.

### Culture of *Cronobacter sakazakii*

The bacterial strain used in this study was *C. sakazakii* CECT 858, supplied by the Spanish Type Culture Collection (CECT, Valencia, Spain), which corresponds with the strain ATCC 29544 of the American Type Culture Collection and is of clinical origin from a child throat. It is recommended as a reference strain by international standards ISO 22964:2017 and ISO 11133:2014/Amd 2:2020. For the reference stock, the bacteria were fixed to porous rings and stored in cryovials at − 80 °C. To cultivate *C. sakazakii*, we followed the procedure described in our previous study (Abad et al. 2022).

### In vitro gastrointestinal digestion

The digestion process used followed the InfoGest Consensus Method and it was based on the protocol by Mackie and Rigby (2015). First, the simulated digestion solutions were prepared: salivary solution (SSS) at pH 7, gastric solution (SGS) at pH 3 and intestinal solution (SIS) at pH 7. These solutions were elaborated according to the concentrations of salts indicated in Table 1 and sterilized with a 0.22 µm low binding protein (LBP) Millipore filter (Merck KGaA, Darmstadt, Germany).

**Table 1:** Composition for a final volume of 200 mL of the simulated salt solutions for the different stages of in vitro GIT digestion (1.25 × concentrations) Adapted from Mackie and Rigby (2015)

Salt	Stock concentration		Simulated solutions					
			SSS pH 7		SGS pH 3		SIS pH 7	
			Vol.	Conc. in SSS	Vol.	Conc. in SGS	Vol.	Conc. in SIS
	g/L	mol/L	mL	mM	mL	mM	mL	mM
KCl	37.7	0.5	7.55	15.1	3.45	6.9	3.4	6.8
KH_2_PO_4_	68	0.5	1.85	3.7	0.45	0.9	0.4	0.8
NaHCO_3_	84	1	3.4	13.6	6.25	25	21.25	85
NaCl	117	2	–	–	5.9	47.2	4.8	38.4
MgCl_2_(H_2_O)_6_	30.5	0.15	0.25	0.15	0.2	0.12	0.55	0.33
(NH_4_)_2_CO_3_	48	0.5	0.03	0.06	0.25	0.5	–	–

*SSS* simulated salivary solution; *SGS* simulated gastric solution; *SIS* simulated intestinal solution

After preparation of all solutions, a static in vitro digestion of dairy formulas was carried out. For this, an initial volume of 4 mL of the corresponding sample was taken, to which 3.2 mL of SSS, 20 µL of CaCl_2_(H_2_O)_2_ and 780 µL of milli-Q water were added, giving rise to a final volume of 8 mL that was adjusted to pH 7. This first step of salivary digestion was incubated for 2 min at 37 °C under agitation. After this time, a 4 mL aliquot of this sample was taken, which was called salivary digest (SD) and it was frozen in liquid nitrogen. The remaining volume was subjected to the next phase of the digestion.

For gastric digestion, 3 mL of SGS, 2 µL of CaCl_2_(H_2_O)_2_, 0.8 mL of porcine gastric pepsin (Sigma Aldrich, St Louis, MO, USA) at a concentration of 2000 U/mL and 118 µL of milli-Q water were added. The final volume was adjusted to pH 3 and incubated for 2 h at 37 °C under agitation. After incubation, a 4 mL aliquot of this sample was removed and frozen in liquid nitrogen to inactivate the effect of the enzymes. This aliquot was called gastric digest (GD). The remaining volume was subjected to the last stage of digestion.

To carry out intestinal digestion, 2.2 mL of SIS, 8 µL of CaCl_2_(H_2_O)_2_, 1 mL of pancreatin (Sigma-Aldrich, 8 × USP) to achieve 100 U/mL of trypsin activity in the final mixture, 0.5 mL of 10 mM porcine bile (Sigma-Aldrich) and 262 µL of milli-Q water were added. The mixture was adjusted to pH 7 and incubated for 2 h at 37 °C under agitation. At the end of the incubation, the entire sample was frozen in liquid nitrogen. This fraction was called intestinal digest (ID).

The three fractions obtained in the digestion were lyophilized and subsequently resuspended with the adequate volume of milli-Q water to obtain a final LF concentration of 5 mg/mL. The digests were filter sterilized using a 2 µm prefilter and a 0.45 µm LBP filter. Additionally, the original undigested formulas were also filtered for later use in the assays. In the case of F1 and F2, centrifugation at 3000 and 5000 g, respectively, for 5 min was required to eliminate some aggregates that made difficult the filtering. Formulas F3 to F6 were passed directly through 2 µm prefilter and 0.45 µm LBP filter. All the samples were stored at − 20 °C until later use.

#### Trypsin activity assay

This analysis, described by Brodkorb et al. (2019), was carried out to assess the activity of the trypsin present in the pancreatin used in the intestinal phase of the in vitro digestion. For this assay, 10 mM p-toluene-sulfonyl-l-arginine-methyl-ester (TAME) (Sigma-Aldrich) was used as trypsin substrate. The reaction of TAME in the presence of trypsin produces p-toluene-sulfonyl-l-arginine and methanol. The objective of the test was to measure the absorbance increase over time to determine the hydrolytic activity of trypsin per min. For this, a solution of trypsin at different concentrations: 10 µg/mL, 15 µg/mL and 20 µg/mL in 1 mM HCl and 46 mM Tris–HCl buffer with 11.5 mM CaCl_2_, pH 8.1, were prepared. First, 2.6 mL of Tris–HCl buffer was mixed with 300 µL of TAME and 100 µL of trypsin and then, continuous absorbance measurement was performed at 247 nm for 10 min. To measure the absorbance, the model 6505 UV/Vis spectrophotometer (Jenway, Stone, UK) was used. Subsequently, the same test was carried out with the pancreatin (1 mg/mL) used in the GIT digestion. The amount of pancreatin used in the digestion assays was based on the activity of the trypsin present in it.

#### Determination of the degree of hydrolysis after digestion

After digestion, the degree of hydrolysis of LF and the proteins in the dairy formulas was determined by the 2,4,6-trinitrobencenesulfonic acid (TNBS) method (Thermo Fisher Scientific, Rockford, IL, USA) based on the protocol by Spellman et al. (2003). TNBS was diluted to 0.1% (w/v) in distilled water. The standards were made with l-leucine (Sigma-Aldrich) at different concentrations, between 15 and 250 µM. In the case of the formulas, gastric and intestinal digests were adequately diluted to ensure that the values obtained fell within the l-leucine standard curve. The digests and standards were diluted in 1% (w/v) SDS and they were analyzed in duplicate. First, 0.25 mL of each sample was added to a tube with 2 mL of 0.2 M sodium phosphate buffer, pH 8.2. Then, 2 mL of TNBS reagent was added. Mix was incubated at 50 °C for 1 h in darkness. After incubation, and to stop the reaction, 4 mL of 0.1 N HCl was added to each tube and allowed to cool for 30 min. After this time, absorbance of the samples was measured at 340 nm.

### Sodium dodecyl sulphate polyacrylamide gel electrophoresis

The protein profiles of the formulas and their digests were analyzed by sodium dodecyl sulphate polyacrylamide gel electrophoresis (SDS-PAGE), using 4–20% polyacrylamide gels, which were stained with Coomassie Blue according to standard procedures. The samples to be characterized were diluted in a 1:1 (v/v) ratio with Laemmli buffer (Laemmli 1970). The molecular weight marker used was the Page Ruler Prestained Protein Ladder (GE Healthcare, Buckinghamshire, UK).

### Antibacterial activity assay against *Cronobacter sakazakii*

In this assay, the antibacterial activity of LF and dairy formulas, before and after different stages of digestion, against *C. sakazakii* was analyzed. First, an isolated colony of this bacterium, was cultured in 10 mL of TSB with 0.6% YE and incubated between 18 and 20 h at 37 °C. The bacterial suspension obtained in stationary phase of growth was diluted in 1% peptone water to an approximate concentration of 10^5^ cfu/mL.

In a sterile 96-well plate, 100 µL of the dairy samples were seeded with 100 µL of *C. sakazakii* suspension. As bacterial growth control, 100 µL of peptone water was added to 100 µL of the bacterial suspension. All samples were analyzed in duplicate in three independent experiments.

The plate was incubated at 37 °C for two incubation times, 4 and 24 h, at which antibacterial activity was assessed by taking 100 µL from each well and making several dilutions that were seeded on TSA plates. The plates were incubated for 24 h at 37 °C to subsequently count the colonies.

### Statistical analysis

In this study, results are presented as the mean ± standard deviation. Statistical analysis of results was performed using the statistical software GraphPad Prism v8.0.2 (GraphPad Software, San Diego, CA, USA). The normality of data was verified with the Shapiro–Wilk test. For data that followed a normal distribution, analysis of variance (ANOVA) was used to compare the means of three or more unpaired groups, and Dunnet’s test was used as a multiple comparison test. Data that did not follow a normal distribution were subjected to the non-parametric Kruskal–Wallis test followed by Dunn's test as a multiple comparison test. Differences with a p value ≤ 0.05 were considered statistically significant.

## Results and discussion

### Degree of protein hydrolysis after in vitro GIT digestion

**Fig. 1:**
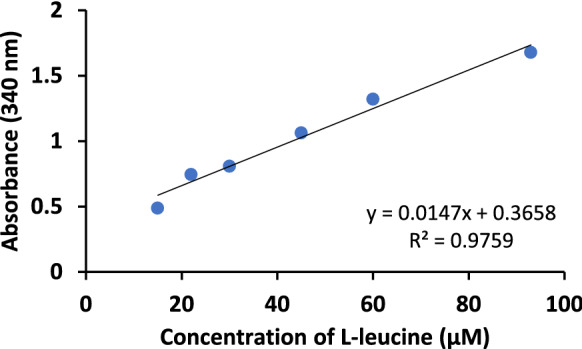
l-leucine standard curve to determine the degree of hydrolysis by TNBS method

The degree of hydrolysis of milk samples after digestion was determined by the method of Spellman et al. (2003). For this, the TNBS reagent was used, which reacts with the amino acids released in the digestion process. Standards were made with l-leucine solutions at concentrations between 0 and 250 µM (Fig. 1) analyzing their absorbance at 340 nm. The values of hydrolysis obtained for the digested samples were referred to those of l-leucine.

The results of the degree of hydrolysis of native LF and dairy formulas after the three stages of digestion are shown in Table 2. Each sample was analyzed in duplicate and an average absorbance value was obtained, which was taken to the standard curve for l-leucine (Fig. 1) to determine the concentration of free amino acids present in each digest as l-leucine amino equivalents. The increase in absorbance at 340 nm is due to an increase in free amino acids release and a higher degree of hydrolysis.

**Table 2:** Degree of hydrolysis of LF and dairy formulas (F1–F6) from each stage of static in vitro GIT digestion expressed in l-leucine amino equivalents

Sample	l-leucine amino equivalent concentration (µM)		
	SD	GD	ID
LF	13.77 ± 3.51	83.02 ± 0.59	94.86 ± 0.23
F1	92.64 ± 1.77	194.70 ± 1.08	446.90 ± 0.53
F2	98.24 ± 0.24	120.29 ± 0.24	494.74 ± 1.90
F3	86.56 ± 1.90	144.78 ± 1.11	431.00 ± 1.90
F4	97.98 ± 0.12	198.08 ± 0.25	428.99 ± 0.94
F5	92.92 ± 2.35	152.87 ± 1.00	424.33 ± 0.17
F6	97.64 ± 0.36	161.05 ± 0.11	421.17 ± 0.75

Values represent the mean ± standard deviation of two replicates in one or two independent experiments (n ≥ 2)

*SD* salivary digest, *GD* gastric digest, *ID* intestinal digest

The free amino acid concentration of the SD was considered basal, since the SSS did not contain any protease and consequently, hydrolysis should not have occurred. Comparing the concentration values of free amino acids of the GD with the basal values, an increase of between 1.2 and 1.7 times was observed, except for LF, which was 6 times higher. It is possible that the amount of pepsin present was not sufficient to hydrolyze the high protein content of dairy formulas. The concentration of free amino acids in the ID showed a higher increase than that of GD with respect to basal values, between 3 and 7 times higher. In the study by Corrochano et al. (2019) the TNBS method was also used to analyze the degree of hydrolysis of bovine whey proteins. Their results indicated that the degree of hydrolysis was greater for a pure protein, such as albumin, than for a whey protein isolate. This conclusion agrees with our study, in which the degree of hydrolysis of pure LF was higher than that of dairy formulas, probably due to a certain degree of protection exerted between proteins in the formulas.

### Antibacterial activity against *Cronobacter sakazakii*

**Fig. 2:**
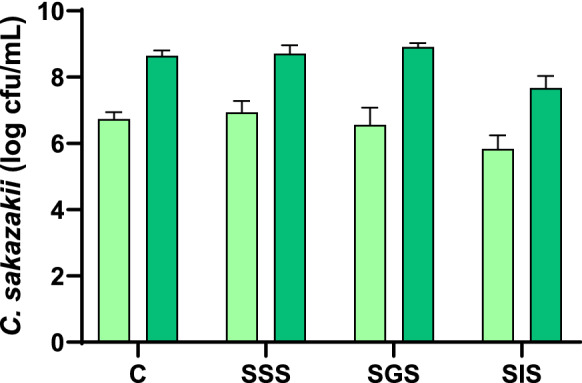
Antibacterial effect of simulated digestion solutions against *C. sakazakii* (light green 4 h; dark green 24 h)*.* *C* Control, *SSS* simulated salivary solution, *SGS* simulated gastric solution, *SIS* simulated intestinal solution. Values represent the mean ± standard deviation of two replicates in three independent experiments (n = 6). (Color figure online)

The first step before analyzing the antibacterial activity of the samples and their digests was to verify that the simulated solutions (SSS, SGS and SIS) used for the in vitro digestion process did not present antibacterial activity against *C. sakazakii*. It was shown that these solutions did not have a significant antibacterial effect against this bacterium (Fig. 2) and, consequently, the activity of the dairy formula digests would be only due to the action of their components and in no case to the solutions used for digestion.

In our previous studies, we have demonstrated that native LF had an antibacterial effect against *C. sakazakii* (Harouna et al. 2015; Abad et al. 2022). In the study by Harouna et al. (2015), it was shown that iron-saturated LF did not have antibacterial activity against this pathogen; therefore, the antibacterial effect of native LF was probably due to its ability to sequester iron from the medium. Furthermore, in the study by Abad et al. (2022) we showed that native LF was effective against *C. sakazakii* both in the exponential and in the stationary phase of growth.

**Fig. 3:**
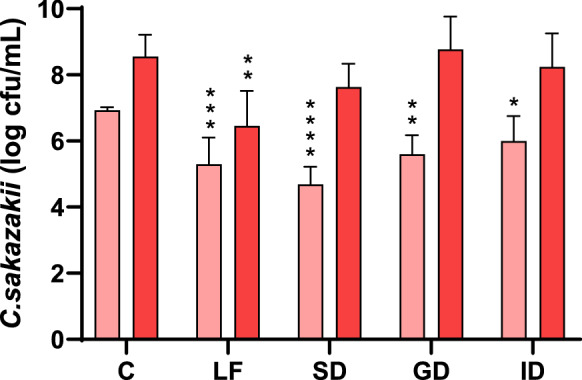
Antibacterial effect of LF and its digests against *C. sakazakii* (light red 4 h; dark red 24 h)*.* *C* Control, *LF* LF at 5 mg/mL, *SD* LF salivary digest, *GD* LF gastric digest, *ID* LF intestinal digest. Values represent the mean ± standard deviation of two replicates in three independent experiments (n = 6). Asterisks indicate significant differences respect to control (*p < 0.05, **p < 0.01, ***p < 0.001, ****p < 0.0001). (Color figure online)

The antibacterial effect of native LF and its digests, obtained after in vitro GIT digestion, was analyzed against *C. sakazakii,* in stationary phase of growth, at 4 and 24 h of incubation (Fig. 3). The antibacterial activity of LF against the bacteria at 4 h of incubation was maintained after all stages of GIT digestion, which could be due to peptides generated in the gastric and intestinal digests, since LF did not appear as the whole molecule in SDS-PAGE. These results coincide with previous studies (Bellamy et al. 1992; Van der Kraan et al. 2004), in which some peptides derived from bovine LF, such as lactoferricin and lactoferrampin, showed antibacterial activity. However, although the antibacterial activity was maintained, it decreased as the digestion progressed. With the action of pepsin, the iron-binding centers of LF could be disrupted, resulting in free iron in the medium and fragments derived from LF unable to bind it (Hopp et al. 2022). Thus, as the digestion stages progress, there will be more iron available to favour the growth of *C. sakazakii*, reversing the possible antibacterial effect of bioactive peptides.

After 24 h of incubation, it was observed that bacteria recovered the ability to grow reaching a level similar to that of the control (Fig. 3); therefore, the effect observed at 4 h was bacteriostatic and then, the bacteria probably developed some mechanisms to counteract the antibacterial activity of LF and their peptides. Digestion does not result in complete hydrolysis of all proteins, and consequently, although in this process peptides are generated and iron is released, it is possible that some LF-derived fragments maintain intact the iron-binding center (Hopp et al. 2022). Faced with the bacteriostatic effect caused by the lack of iron, the bacteria may develop defense mechanisms, including the production of siderophores. These siderophores could sequester iron from the medium and from LF fragments, forming stable complexes with it (Phair et al. 2022) and allowing the bacteria to recover.

**Fig. 4:**
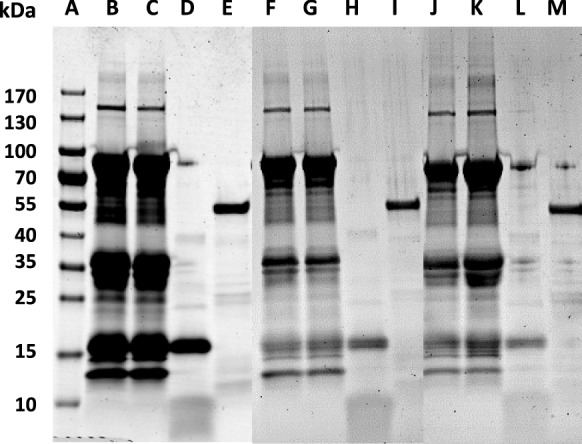
SDS polyacrylamide gel electrophoresis (4–20%) of digests of whey-based formulas (F1, F3 and F5) stained with Coomassie Blue. (A) Molecular weight marker, (B) F1, (C) F1 salivary digest, (D) F1 gastric digest, (E) F1 intestinal digest, (F) F3, (G) F3 salivary digest, (H) F3 gastric digest, (I) F3 intestinal digest, (J) F5, (K) F5 salivary digest, (L) F5 gastric digest, (M) F5 intestinal digest

Similarly, the antibacterial activity of the different dairy formulas (F1-F6) and their digests was also evaluated at 4 and 24 h. After digestion of these formulas, the extracts from each stage were analyzed by electrophoresis to determine their composition (Fig. 4). It was observed that the salivary phase had no effect on the proteins, as it was expected since the SSS did not contain proteases. In the gastric digests, some changes were observed due to the proteolytic activity of pepsin, showing bands, corresponding to peptides of 10 kDa or smaller (lanes D, H and L in Fig. 4). In the intestinal digest several bands were observed, corresponding to lipase (55 kDa) and several proteases, but the peptides that appeared in the gastric phase disappeared (lanes E, I and M in Fig. 4). This may be due to the action of pancreatin proteases, such as trypsin, that continue the hydrolysis of those small peptides. The peptides obtained after intestinal digestion were probably so small that they were not retained in the electrophoresis gel. This electrophoretic pattern was very similar in all samples regardless of the dairy formula considered.

De Figueiredo Furtado et al. (2021) performed GIT digestion of some infant formulas based on whey protein isolate or whey protein hydrolysate, supplemented with LF, carbohydrates and fatty acids. In their study, α-LA was almost completely hydrolyzed in the gastric phase. However, β-LG resisted the gastric phase of digestion and it was not proteolyzed until it was subjected to the intestinal phase, in which all remaining proteins were hydrolyzed into small peptides. These results agree with those observed in our work when analyzing the formula digests by electrophoresis (Fig. 4).

In the study by De Figueiredo Furtado et al. (2021), LF was resistant to the gastric phase and was hydrolyzed in the intestinal stage. In our case, LF was mainly hydrolyzed in the gastric phase, generating bioactive peptides in this stage. In the study by De Figueiredo Furtado et al. (2021) the iron saturation of LF was not specified. Therefore, the difference in the digestion stage, in which LF is hydrolyzed, could be due to the fact that their LF is more saturated than our LF, which has an iron-saturation below 10%. It is well known that the structure of iron-saturated LF is more compact than that of the protein devoid of iron and, therefore, more resistant to hydrolysis (Sánchez et al. 1992). The different results of these two studies may also be explained by the difference in pH at the stages of digestion. In our study, the gastric stage was carried out at pH 3, which can cause that some bonds of LF molecule start to break, thus facilitating its hydrolysis. At that pH, some bonds that stabilize LF structure begin to break (Sánchez et al. 1992). However, it is possible that in the study by De Figueiredo Furtado et al. (2021) the gastric phase did not reach pH 3, and LF resisted more to enzymatic activity.

**Fig. 5:**
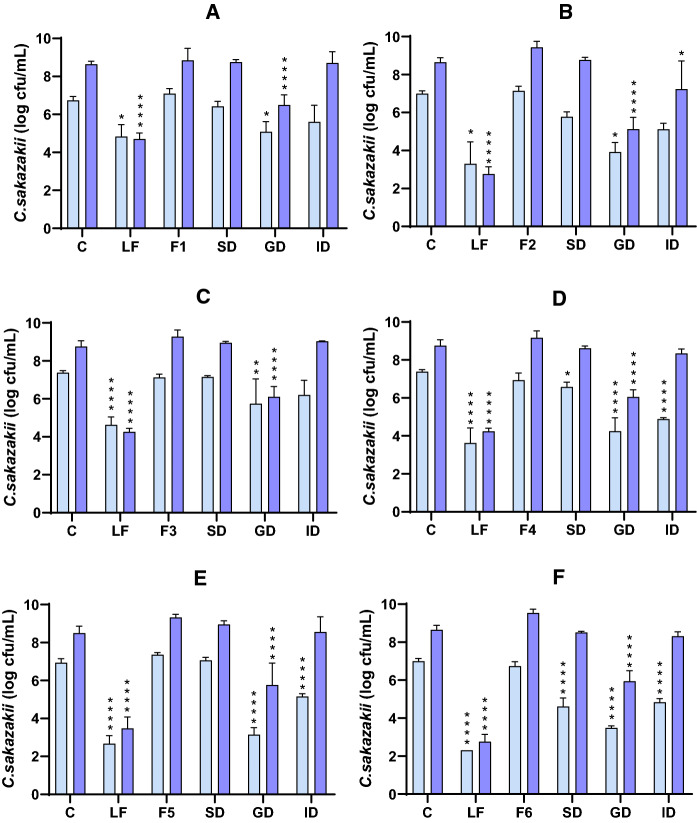
Antibacterial effect of **A** F1 and its digests, **B** F2 and its digests, **C** F3 and its digests, **D** F4 and its digests, **E** F5 and its digests, and **F** F6 and its digests against *C. sakazakii* (light purple 4 h; dark purple 24 h)*.* *C* Control, *LF* LF at 5 mg/mL, *SD* salivary digest, *GD* gastric digest, *ID* intestinal digest. Values represent the mean ± standard deviation of two replicates in, at least, two independent experiments (n ≥ 4). Asterisks indicate significant differences respect to control (*p < 0.05, **p < 0.01, ****p < 0.0001). (Color figure online)

The results of the antibacterial activity of dairy formulas and their digests are shown in Fig. 5. Mostly, formulas before digestion and the digests of the salivary phase did not show antibacterial activity, except for the SD of F4 and F6, both formulas based on buttermilk and subjected to a technological treatment. These digests did show an effect against *C. sakazakii* at 4 h of incubation (Fig. 5D, F). However, the highest activity was presented by the GD, being higher in the formulas subjected to thermal treatment, F5 and F6 (Fig. 5E, F). This antibacterial activity decreased after intestinal digestion, allowing greater bacterial growth, possibly due to the effect of proteolysis on the bioactive peptides.

LF at 5 mg/mL is shown as a positive control of the antibacterial effect. Some differences can be observed in LF activity between the different graphs (Fig. 5A–F), since all the tests were carried out independently, which could generate a small variation in its effect. It is important to point that LF used and the procedure followed for its preparation were the same in all assays. Although the bacteria came from the same stock and used always in the stationary phase, there could have been some variability between the experiments in the bacterial metabolic activity and susceptibility to LF. In any case, LF always showed great antibacterial activity, decreasing bacterial counts by 2–4 logarithmic units.

In addition, it is worth highlighting the difference between the effect of LF and the different formulas, the native protein always being the one that showed the greatest effect. According to the literature, this lower effect of LF when it is part of a dairy formula compared with LF alone in its native state could be due to the interaction with other proteins or components, like β-LG, albumin (Lampreave et al. 1990) or casein micelles (Anema 2018), its effect being reduced because of its lower availability.

Comparing the activity of F1, based on whey, and F2, based on buttermilk (Fig. 5A, B), a slightly greater antibacterial activity of F2 was observed after the gastric phase. This effect could be explained by the high content of MFGM, since F2 contains apart from that added to the formula, the MFGM naturally present in buttermilk. Additionally to bioactive proteins with antimicrobial properties present in MFGM (Parrón et al. 2018), other components such as glycosphingolipids and phospholipids are also being investigated, as they function as intracellular signalling molecules in a wide variety of biological processes (Fuller et al. 2013). For this reason, in recent years, the addition of MFGM, or fractions enriched in it, to infant formulas has been done in certain products.

In the case of F3 and F4, the added MFGM was previously subjected to homogenization, which caused some small differences in the results (Fig. 5C, D). As expected, it was observed that after the salivary phase there was no hydrolysis of the proteins and the SD of F3, based on whey, had no activity against the pathogen. However, F4 based on buttermilk, showed some antibacterial activity after the salivary phase, with little significant difference respect to the control. This could be because during homogenization, fat globules decrease their size and increase their surface, releasing the original MFGM proteins and favouring the binding of caseins to the membrane (Lee and Sherbon 2002) and also of LF, as it has been reported that LF binds naturally to casein micelles (Anema 2018). Therefore, the binding of LF to the homogenized MFGM could hinder its antibacterial ability. However, according to this argument, the MFGM proteins released in F4 could exert their activity.

On the other hand, GDs of F3 and F4 were effective at 4 and 24 h of incubation, with GD of F4 having greater antibacterial activity, producing greater decrease in the growth of *C. sakazakii*. The antibacterial activity of F3 and F4 decreased after the intestinal phase, the ID of F4 being the only one that maintained activity only at 4 h of incubation. Therefore, it can be concluded that the F4 digests, composed of homogenized buttermilk, had greater activity against *C. sakazakii* than the F3 digests based on whey.

Homogenization improves the digestibility of proteins (Tunick et al. 2016), facilitating the release of bioactive peptides. Furthermore, MFGM has a great amount of bioactive proteins, such as lactadherin, butyrophilin and mucin, with antibacterial effect (Lönnerdal 2016) and it has been shown that the antibacterial activity of some MFGM hydrolysates may be due to the generation of biodefensive peptide sequences (Clare et al. 2008). In our study, F4 had more effect than F3, which could be due to the higher MFGM content of the former.

Formulas F5 and F6 were subjected to a thermal pasteurization treatment of 72 °C for 20 s. The SD of F5 did not show antibacterial activity, while the SD of F6 showed some effect, with significant differences respect to the control at 4 h of incubation (Fig. 5E, F). The digests of F5 and F6 from the gastric phase showed antibacterial activity, with significant differences at 4 and 24 h, decreasing the growth of the pathogen by up to 4 log cfu/mL. The antibacterial effect of the ID of F5 and F6 was maintained at 4 h, although with less activity than the GD, and disappeared after 24 h of incubation.

The results obtained indicate that heat treatment has positive effects on the antibacterial activity of dairy formulas, possibly because it favours the release of active peptides. In the study by Halabi et al. (2020) it was observed that the application of thermal treatment to infant formulas made with whey increased the susceptibility of some proteins to pepsin hydrolysis. The resistance of α-LA and β-LG to hydrolysis was not modified by heating, while LF increased its susceptibility compared to the unheated form. The results obtained in that study also indicated that the kinetics of LF hydrolysis in intestinal digestion of heat-treated infant formulas were higher than those of untreated ones. The loss of activity of denatured and hydrolyzed LF could be compensated by the release of peptides with high antibacterial activity.

We can affirm from our results that dairy formulas showed antibacterial activity against *C. sakazakii* when were subjected to static in vitro digestion. In all the dairy formulas, the SD did not have antibacterial action, while the highest activity was observed in the digests of the gastric phase and it was maintained, to a greater or lesser extent, after the intestinal phase. There was greater antibacterial effect against the pathogen at 4 h of incubation than at 24 h, especially in the intestinal phase. In addition, it was observed that the formulas based on buttermilk (F2, F4 and F6) had greater antibacterial effect than those based on whey (F1, F3 and F5). This could be due to the effect of the proteins present in the MFGM, which were found in greater proportion in the formulas based on buttermilk. The XO, one of the main components of this membrane, generates H_2_O_2_ via an enzymatic reaction, which can cause bacterial death (Clare et al. 2008). Furthermore, it was observed that these biodefensive properties of XO were preserved after proteolysis (Clare et al. 2008). Likewise, the thermally treated formulas (F5 and F6) showed greater activity than those treated by homogenization (F3 and F4), and also than those with no treatment (F1 and F2), mainly in the gastric phase. It was also observed that heat treatment seemed to have a beneficial effect on formula F5, as it was the only formula based on whey with the highest activity against *C. sakazakii*.

In short, the GD presented the highest antibacterial activity against *C. sakazakii* in all formulas. This activity may be due to the release of bioactive peptides by the enzymatic proteolysis that occurs during digestion. After hydrolysis, proteins can become bioactive, increasing their inhibitory effect on pathogens (McEvoy et al. 2016).

**Fig. 6:**
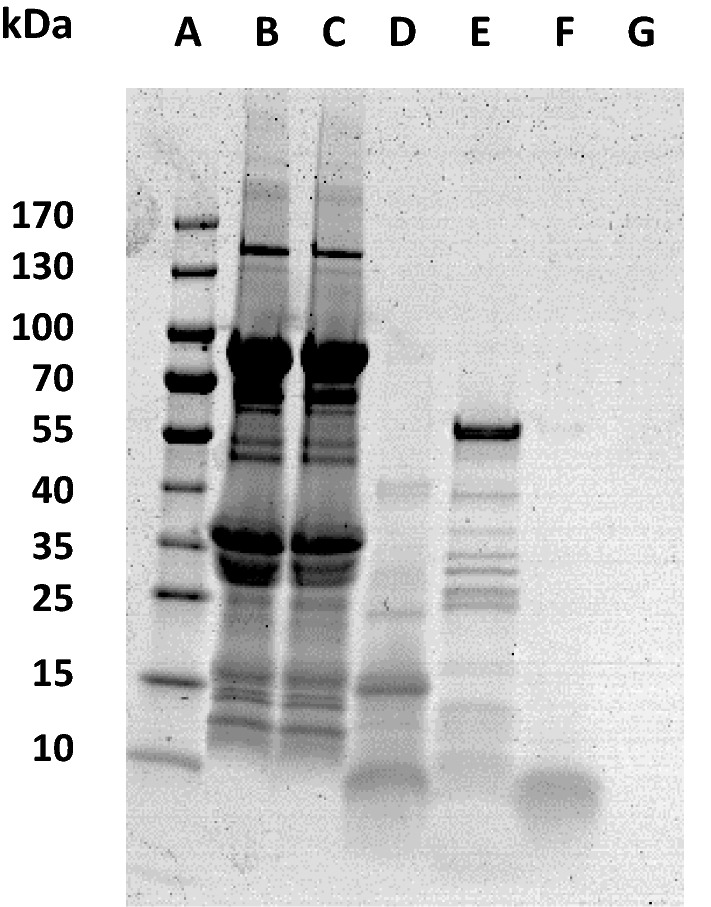
SDS polyacrylamide gel electrophoresis (4–20%) of digests of F2 stained with Coomassie Blue. (A) Molecular weight marker, (B) F2, (C) F2 salivary digest, (D) F2 gastric digest, (E) F2 intestinal digest, (F) 3–10 kDa fraction, (G) less than 3 kDa fraction

To characterize the peptides generated after gastric digestion and to find out the molecular weight of those that were mainly responsible for the antibacterial action, the GD of one formula was fractionated. Since the digestion of all the formulas followed the same procedure and generated a similar electrophoretic pattern of hydrolysates (as shown in Fig. 4), the fractionation was performed only with one gastric digest. To make this selection, an untreated formula was preferred, since technological treatment could have generated protein–protein interactions and aggregates that would make more difficult the separation of peptides. Finally, F2 was chosen because it presented higher antibacterial activity. After fractionation, peptides with a size between 3 and 10 kDa and less than 3 kDa were differentiated (Fig. 6).

In the lane corresponding to the fraction of peptides between 3 and 10 kDa (lane F), a band was observed at the lowest part of the gel, while in the fraction of peptides smaller than 3 kDa (lane G) no band was observed, probably because they are not retained in the gel due to their small size.

To complete the analysis, the antibacterial activity of the two fractions was evaluated (Table 3). It was observed that both had antibacterial effect and that the fraction between 3–10 kDa had greater activity than the fraction lower than 3 kDa. The 3–10 kDa fraction maintained its antibacterial activity at 24 h, while the fraction lower than 3 kDa significantly decreased its effect.

**Table 3:** Antibacterial activity of the fractions obtained from gastric digest of F2 by ultrafiltration

Incubation of 4 h		Incubation of 24 h	
3–10 kDa peptides		3–10 kDa peptides	
Mean ± SD	4.09 ± 0.36****	Mean ± SD	5.34 ± 0.31****
Percentage	59.1	Percentage	65.1
< 3 kDa peptides		< 3 kDa peptides	
Mean ± SD	5.42 ± 0.03****	Mean ± SD	7.44 ± 0.36*
Percentage	78.3	Percentage	90.7

Values represent the mean ± standard deviation of *C. sakazakii* log cfu/mL from two replicates in two independent experiments (n = 4) and are also expressed as a percentage respect to the control (*p < 0.05, ****p < 0.0001)

The GIT digestion of food has great influence on the release of peptide sequences present in the proteins. The released peptides can carry out several biological functions, in the intestinal lumen and in the different organs of the body if they are absorbed and transferred to systemic circulation (Aspri et al. 2018). In the study by Aspri et al. (2018), fermented donkey milk showed activity against *Listeria monocytogenes* before digestion. After in vitro GIT digestion, its antibacterial activity against that pathogen increased. This enhanced antibacterial activity was also detected against *Staphylococcus aureus* and *Bacillus cereus*. The high level of LF and lysozyme contained in donkey milk could be, in part, responsible for the high antibacterial activity observed. Furthermore, these authors pointed that donkey milk fermented with lactic acid bacteria had different protective factors with antimicrobial activity, including the peptides released during the process of digestion. These factors can have an impact on intestinal health, especially beneficial for the immune defence system of children and elderly individuals.

In the study by Nielsen et al. (2018), 58 bioactive peptides were released during gastric digestion of proteins from human and bovine milk in premature infants. These peptides presented sequences closely related to known peptides with different activities: antioxidant, antithrombotic, antimicrobial, etc. The results of Nielsen et al. (2018) and those of the present study indicate the great interest in investigating bioactive peptides derived from milk proteins, as potential ingredients of functional foods.

## Conclusions

Dairy by-products have an interesting potential as they contain bioactive compounds. Among these compounds, lactoferrin and the milk fat globule membrane have great value, and would allow the revaluation of those dairy by-products. However, it is necessary to know the effect of technological treatments when bioactive compounds are incorporated into products for consumption and also the effect of gastrointestinal digestion on their activity. The results obtained in this study show that LF maintains its antibacterial activity against *C. sakazakii* after being subjected to static in vitro digestion. In addition, it has been observed that dairy formulas containing bioactive proteins have antibacterial activity after being digested, especially after the gastric phase. Furthermore, it has been observed that pasteurization has positive effects on their antibacterial activity, possibly due to the partial denaturation of some proteins that facilitates the action of digestive proteases and the release of bioactive peptides.

In conclusion, this study provides useful information on dairy by-products, such as whey and buttermilk, and the effect of digestion and technological treatments on them; being potential ingredients for functional products directed to adults.

In the future, it would be necessary to carry out similar studies subjecting dairy formulas to digestion in different conditions, taking into account the variations that the digestion process may have in the infant’s digestive tract, depending on age and intestinal maturation. This could extend our knowledge of how to use these by-products in the preparation of infant formulas and functional products.
